# Evaluation of MPEG-4 AAC Audio Codec Using EEG and EMG Signals for Use with DICOM® Neurophysiology

**DOI:** 10.1007/s10916-026-02451-9

**Published:** 2026-08-26

**Authors:** Mattia Galanti, Filippo Battaglia, Giovanni Gugliandolo, Petter M. Omland, Kristian B. Nilsen, Sarah Breevoort, M. Noreen Herring, Jerome Kurent, I-Hweii A. Chen, Tuan Vu, Kyle Wuthrich, Nicola Donato, Mulugeta Gebregziabher, Giuseppe Campobello, Brian C. Dean, Jonathan J. Halford

**Affiliations:** 1 School of Computing, Clemson University, Clemson, 29631 SC USA; 2 Department of Engineering, University of Messina, Messina, 98166 Italy; 3 Department of Neuromedicine and Movement Science, Faculty of Medicine and Health Sciences, Norwegian University of Science and Technology, Trondheim, N-7491 Norway; 4 Department of Neurology, Section of Clinical Neurophysiology, Oslo University Hospital, Oslo, Norway; 5 Ralph H. Johnson Veterans Affairs Medical Center, Charleston, 29401 SC USA; 6 Department of Neurology, Medical University of South Carolina, Charleston, 29425 SC USA; 7 Morsani College of Medicine, University of South Florida, Tampa, 33602 FL USA; 8 Department of Public Health Sciences, Medical University of South Carolina, Charleston, 29425 SC USA

**Keywords:** Data compression, EEG, EMG, MPEG-4 AAC, Neurophysiology

## Abstract

Modern neurophysiological recording technologies are producing large volumes of data. This study investigates the use of the Moving Picture Experts Group version 4 Advanced Audio Coding (MPEG-4 AAC) for compressing electroencephalography (EEG) and electromyography (EMG) signals. All EEG signals contained either seizures or interictal epileptiform discharges (IEDs), which are nonstationary patterns characterized by sharp transients. Differences between pairs of original single-channel signals and compressed/decompressed signals were explored using the Percentage Root Mean Square Difference (PRD), power spectral density (PSD) features, and clinical expert opinion of waveform morphology. PRD was used as the primary error measure and PSD features were explored across different frequency bands. The results showed substantial decline in signal quality based on human expert opinion for compressed then reconstructed EEG signals with PRD error greater than 15% and EMG signals with PRD greater than 1%. PSD analysis showed significant changes in the gamma and beta bands for both EEG and EMG signals. This study provides further evidence that standard audio codecs which use psychoacoustic models, such as MPEG-4 AAC, do not sufficiently support compression of neurophysiology waveforms.

**Supplementary Information:**

The online version contains supplementary material available at 10.1007/s10916-026-02451-9.

## Introduction

Electroencephalography (EEG) is a technique that is both non-invasive and cost-efficient for examining neurophysiological functions. It involves placing electrodes on the scalp to capture the electrical activity generated by large groups of neurons firing in synchrony in the brain [1]. EEG is primarily employed to evaluate and manage patients with seizures and sleep disorders, but is also used to evaluate brain function in neurodegenerative diseases like Alzheimers, encephalopathies, and brain injuries [2–4]. Electromyography (EMG) is a procedure that involves recording the electrical impulses generated by muscle tissues during neuromuscular activity [5]. EMG is a critical tool for identifying neuromuscular disorders such as amyotrophic lateral sclerosis (ALS) and muscular dystrophy, and it can help clinicians differentiate between muscle-related conditions and nerve-related disorders [6–8].

Technological advancement in brain signal recording and processing, such as high-density multi-electrode arrays (HD-MEAs) and amplifiers with large numbers of input channels and high sampling rates, are producing biomedical signal datasets with unprecedented spatial and temporal resolution, advancing our understanding of brain processes and neuronal interactions [9]. Due to the size of these datasets, data storing and forwarding is becoming ever more challenging. Efficient data handling is crucial for future big data technologies such as deep learning algorithms.

Data compression is essential to minimize storage requirements and transmission bandwidth while preserving the integrity of recorded signals. Memory limitations pose a significant challenge, as storing vast amounts of high-resolution data can be prohibitive without adequate compression [10, 11]. Data access and retrieval times can also be affected by compression methods [12]. In medical applications, advanced compression techniques can ensure that vital clinical data can be retained and accessed quickly, which is crucial for accurate patient diagnosis and intervention [13].

There is no standard codec for the storage and transmission of biomedical waveform data such as EEG, EMG, and electrocardiography (ECG) signals. Standard audio codecs are probably insufficient to serve as a codec for neurophysiology signals generally because: (1) the set of sampling rates commonly used for audio vary significantly from the typical sampling rates of biomedical waveform recordings; (2) audio codecs usually rely on a psychoacoustic masking model and/or assume specifics about the frequency range of audio signals which does not apply to biomedical waveforms; and (3) such audio codecs are ordinarily not designed to operate in the near-lossless range of quality [14–16].

In this manuscript, we extend our report of audio codec performance for biomedical waveforms by reporting an exploratory study on Moving Picture Experts Group version 4 Advanced Audio Coding (MPEG-4 AAC) performance on clinical EEG and EMG data. Recent work on EEG and intracranial electroencephalography (iEEG) compression has reported Percentage Root Mean Square Difference (PRD)–compression-ratio trade-offs while explicitly including Advanced Audio Coding (AAC) as a baseline [17]. In prior summaries of EEG compression methods, representative studies reported PRD values ranging from below 1% in near-lossless settings to approximately 5%–7% in more lossy regimes across predictive/entropy-based, wavelet-based, and related transform-domain approaches, with more recent deep learning-based methods showing operating-point-dependent distortion behavior. More broadly, EEG and EMG compression research includes wavelet- and transform-domain methods, predictive and entropy-based approaches, compressed-sensing strategies, near-lossless techniques, and learning-based reconstruction methods [18, 19]. To provide context for the distortion levels observed with MPEG-4 AAC, Table 1 summarizes representative EEG and EMG waveform compression studies that reported PRD or PRD-equivalent reconstruction error. Because compression metrics, datasets, signal modalities, and operating points vary substantially across studies, the table should be interpreted as a compact literature-derived reference rather than a formal cross-method benchmark. An additional overview of neurophysiology waveform codec performance and Digital Imaging and Communications in Medicine (DICOM®) standardization context is provided by Battaglia et al. [20]. Against this background, our goal was to provide an additional evaluation of this commonly used audio-focused psychoacoustic codec on neurophysiological signals and to determine its usefulness for clinical waveform archiving and reuse. We performed evaluation of EEG and EMG waveform signals before and after MPEG-4 AAC compression/decompression using neurologist expert opinion on the appearance of the waveforms and by comparison of power spectral density (PSD) features. Our results have provided information to the International Telecommunication Union-Telecommunication Standardization Sector (ITU-T), which helped support the recent ITU-T Question 6 / Study Group 16 (Q6/SG-16) Call for Proposals for Biomedical Waveforms Coding [21].

This report is organized as follows: Section “Materials and Methods” covers the core methods of our study. In order, Subsection “Datasets and Codec Application” introduces the datasets and the application of the codec, providing an overview of the data used in our study and explaining how the codec was applied. Subsection “EEGnet Scoring” describes the EEGnet scoring methodology, including the EEGnet framework and its use in scoring and analyzing both EEG and EMG data. Subsection “Data Grouping” details the data grouping process, outlining the criteria and methods used for data grouping. Subsection “Distortion Metrics” explains the metrics used in the study to evaluate the impact of compression on signal distortion. Subsection “Statistical Analysis” presents the statistical analysis, detailing the statistical methods and tests used to validate our findings. Section “Experimental Results” reports the experimental results, including canonical and extended frequency-band analyses for EEG and EMG signals, showcasing key findings and observations. Finally, Section “Conclusions” summarizes the main conclusions of our study and suggests directions for future research.

## Materials and Methods

### Datasets and Codec Application

The EEG data used in this study were obtained from the Medical University of South Carolina (MUSC), and include de-identified, approximately 10-minute excerpts from 41 recordings made with Natus equipment. This collection includes both scalp EEGs recorded with the 10-20 electrode system [22] and intracranial EEGs from depth electrodes. Of the 41 EEG recordings, only 16 were intracranial EEGs; therefore, this exploratory dataset was not intended to support a separate PRD acceptability cutoff for intracranial EEG alone. For our analysis, 150 one-minute segments were selected to represent a broad range of codec-generated distortion levels. The EEG segments included seizures and interictal epileptiform discharges (IEDs), selected to challenge the codec under nonstationary, transient-rich conditions. The EMG dataset comprises multichannel surface EMG research recordings from four forearm muscles of 40 subjects from a freely available database [23]. A set of 145 single-channel signals was chosen to cover a wide range of codec-induced distortion levels. The neurophysiological recordings used in this study were deidentified and non-coded and no demographic or clinical information (such as age or health status) was available to the investigators.

The biometric data were processed using the AAC encoder implementation released by Fraunhofer Institute for Integrated Circuits (Fraunhofer IIS) (FDK AAC) [24], where compression performance was controlled via the “–bitrate-mode” parameter. To satisfy the minimum sampling rate requirement of MPEG-4 AAC, a workaround was employed by specifying fake compression frequencies. Detailed descriptions of the electrode configurations, recording settings, selection criteria, and the encoder settings used in this study are provided in the Supplementary Information.

### EEGnet Scoring

The EEGnet framework facilitated the evaluation of signals by experts. EEGnet is an application for viewing and annotating biomedical signals using a web interface [25, 26]. For this study, a unique scoring paradigm was developed whereby pairs of single-channel original and compressed/decompressed EEG and EMG signals were displayed to experts (with original signal displayed above the compressed/decompressed signal). Experts evaluated the signals by answering a binary prompt: “Did you see a clinically significant difference between the two signals?” In this study, “clinically significant distortion” was operationalized as distortion judged by the expert reviewer to meaningfully affect clinical interpretation of the waveform. Each signal received a score ranging from 0 to 8, representing the number of experts who found the distortion unacceptable. For further information on the expert evaluation interface and the scoring methodology, please refer to the Supplementary Information.

### Data Grouping

For the PSD analysis, the EEG signals were categorized into two groups due to the varying sampling rates (which could affect their frequency content and the performance of the codec). Each of the EEG signals has one of the following sampling rates: 256, 512, 1024 or 2048 Hz. Most of the EEG signals present a sampling rate of 512 Hz or less. All EMG recordings have a sampling rate of 2000 Hz. To account for these differences, three groups are defined as follows:

1. EEG recordings with a sampling rate $f_{s} \le 512$ Hz (i.e., 256 or 512 Hz);
2. EEG recordings with a sampling rate $f_{s}>512$ Hz (i.e., 1024 or 2048 Hz);
3. EMG recordings with a sampling rate of 2000 Hz.

It is worth mentioning that Group 1 recordings generally correspond to scalp EEG signals, whereas Group 2 recordings generally correspond to iEEG signals.

**Table 1:** Representative EEG and EMG compression studies reporting PRD-based distortion metrics

Refs.	Signal	Method	Compression	Metric	Metric	Compression	PRD	Notes
	type	class	approach		definition	value	range	
[27]	EMG	Wavelet / entropy coding	DWT + Kohonen NN bit allocation + Huffman coding	CF	CF = (OS – CS) / OS *× * 100	50–85%	Isometric: 2.4–7.0%; isotonic: 0.4–7.2%	Surface EMG from isometric/isotonic contractions. PRD values are reported separately for isometric and isotonic EMG contractions.
[28]	EEG	Wavelet / optimization	Block-based Haar wavelet + COVIDOA coefficient selection	CR	CR = NO / NR	Mean CR 12.6668	Mean PRD 0.4014%	Motor Movement/Imagery EEG dataset. Only EEG results are reported here; ECG results from the same study were not included.
[29]	EMG	Compressed sensing	Bernoulli matrix + db2 sparse basis + BP reconstruction	CR	CR = 1 – M/N	0.1–0.9	23.74 ± 7.82% to 103.48 ± 3.77%	MIT-BIH polysomnographic EMG. Included as an EMG compressed-sensing study; reconstruction error remained relatively high across the reported compression settings.
[30]	EEG	Spatial pseudo-codec	Logarithmic normalization + integer/fractional coding + RLE	CR	Reported as compression ratio	Mean CR 3.16	Mean PRD 2.72%	Eight public multichannel EEG datasets. The paper reports CR directly, but an explicit CR equation was not available.
[31]	EEG	Wavelet / learning-assisted	1D-to-2D mapping + bior4.4 wavelet + STW/EZW/SPIHT/LVL-MMC + adaptive filtering	CR	CR = So / Sc	Zigzag + STW: CR 89.30	PRD 0.23%	HUSM depression EEG dataset.
[32]	EMG	Learning-based compression	Deep convolutional autoencoder; compared with wavelet thresholding and HEVC	CR	CR = original size / compressed size	CR 1600; effective CR 6400 with 6-bit quantization	PRDN 31.5% at CR 1600; PRDN 31.5 ± 0.4% at effective CR 6400	Forearm and HD-EMG recordings. The study reports PRDN; the authors state that PRDN is equivalent to PRD because the DC offset was removed.
[33]	EEG / EMG	Compressed sensing	Bernoulli matrix; DB4 basis for EMG; DCT/DFT decoding for EEG; modified CoSaMP	CR	CR = measurements / original samples	CR 0.4	EEG PRD 9.1%; EMG PRD 21.3%	Included because both EEG and EMG were evaluated using the same compressed-sensing framework.
[34]	EMG	Image-based / JPEG2000	2D EMG matrix + percentage-difference or relative-complexity sorting + JPEG2000	CF	CF = (Bo – Bc) / Bo *× * 100	75–90%	Percentage-difference sorting: 1.46–7.65%; relative-complexity sorting: 1.48–7.74%	Isometric biceps brachii EMG. A single row summarizes the two preprocessing strategies evaluated before JPEG2000 compression.
[35]	EMG	Wavelet/DCT image-based coding	2D EMG matrix; blockwise DWT or DCT; SPIHT coding	CR	CR = 100 *× * (1 – compressed size / original size)	Approx. 60–67% in reported examples/curves	Example PRD 0.01–0.22%; up to about 7% at higher compression settings	Biceps EMG datasets. Included as broader EMG compression context; reporting is less standardized than in the other studies.
[36]	EEG	Deep compressed sensing	OMP reconstruction + CNN-LSTM refinement	Comp. rate %	Definition not explicitly specified	30–70%	6.99–10.62%; best PRD 6.99% at 480 Hz and 70% compression rate	Sleep-EDF-SC single-channel EEG. Included as a recent deep compressed-sensing EEG study; the method is conceptually related to Du et al. [37]
[37]	EEG	Deep compressed sensing	CS-ResNet; compared with OMP, CoSaMP, RNN, CSNet, CS-DRN, and MFF-SE	CR	CR = M/N	10–90%	0.5485–30.2299% across CR 90–10%; 0.5485–1.0016% across CR 90–40%	BCI Competition IV-2a EEG. Included as a recent EEG deep-learning compressed-sensing reference with clearly reported PRD values.
[38]	EEG	Wavelet / entropy coding	2D EEG arrangement + 5-level bior4.4 DWT + quantization + thresholding + adaptive arithmetic coding	CR	CR = L0 / Lc	Graz: CR 5–15; CHB-MIT: CR 11.07	Graz: PRD 5.7% at CR 5 and 20.8% at CR 15; CHB-MIT: PRD 10.44% at CR 11.07	Graz BCI Competition 2008 and CHB-MIT scalp EEG. Included as a wavelet/entropy-coding EEG compression reference using public EEG datasets.
[39]	EMG	Deep compressed sensing	Bernoulli matrix + TCN/full-connection reconstruction; compared with OMP, BP, and modified CoSaMP	CR	CR = M/N *× * 100%	10–90%	TCNN PRD <50% when CR >30%; PRD 15.7% at CR 50%; PRD 9 at mean CR 59.91%	Ninapro DB2 myoelectric signals. The reported CR represents the retained measurement percentage rather than the conventional original-size/compressed-size ratio.

*Abbreviations:* BCI, brain-computer interface; BP, basis pursuit; CF, compression factor; CNN-LSTM, convolutional neural network–long short-term memory; CoSaMP, compressive sampling matching pursuit; COVIDOA, coronavirus disease optimization algorithm; CR, compression ratio; DCT, discrete cosine transform; DFT, discrete Fourier transform; DWT, discrete wavelet transform; ECG, electrocardiography; EEG, electroencephalography; EMG, electromyography; EZW, embedded zerotree wavelet; HD-EMG, high-density electromyography; HEVC, high-efficiency video coding; LVL-MMC, level-wise motion magnitude coding; NN, neural network; OMP, orthogonal matching pursuit; PRD, percentage root-mean-square difference; PRDN, normalized PRD; RLE, run-length encoding; SPIHT, set partitioning in hierarchical trees; STW, spatial-orientation tree wavelet; TCN, temporal convolutional network. *Note:* Compression metrics are reported as defined in each original study and are not necessarily directly comparable across studies

### Distortion Metrics

We evaluated the impact of the distortion introduced by the compression and decompression process in terms of the PRD defined as follows:

1 $$\begin{aligned} \text {PRD} = 100\% \cdot \sqrt{\frac{ \sum _{j=0}^{M-1} (a_{j} - \tilde{a}_{j})^2}{ \sum _{j=0}^{M-1} a_{j}^2}} \end{aligned}$$

where * M * is the number of samples of the input signal, $a_{j}$ is the * j *-th original sample (with * 0 ≤ j < M *) and $\tilde{a}_{j}$ is the corresponding reconstructed sample after bitstream decoding.

Moreover, we evaluated the distortion introduced by the compression on the PSD considering five distinct spectral bands: delta (1–4 Hz), theta (4–8 Hz), alpha (8–13 Hz), beta (13–30 Hz), and gamma (30–100 Hz). These spectral bands were chosen based on a traditional approach to PSD analysis and previous neurophysiological signal analysis research [40]. Because EMG contains substantial spectral energy above 100 Hz, the gamma range was extended for EMG analysis, subdivided into low- and high-gamma (30–100 Hz and 100–400 Hz) to capture its extended frequency content (see Section “Experimental Results”). All EEG and EMG data files were exported in European Data Format (EDF) [41]. The PSD analysis was performed using MATLAB and its Signal Processing Toolbox [42].

To further assess the impact of compression on the frequency content of the signals, we computed the “power of the errors” in each spectral band, which quantifies the discrepancy between the normalized PSD of the original and reconstructed signals. Detailed equations, including the full configuration of MATLAB’s *pwelch()* function [43, 44] and the derivation of the power-of-errors metric, are provided in the Supplementary Information.

### Statistical Analysis

The statistical analysis followed the steps below:

1. **Assess normality.** We assessed normality of the power-of-the-errors distributions $\mathcal {E}_{i,X}$ using the Lilliefors-corrected Kolmogorov–Smirnov test (MATLAB lillietest [45]), which is appropriate when the normal mean and variance are estimated from the same sample. We did not test against a fixed $N(\mu _0,\sigma _0)$. This choice follows standard guidance that lists Kolmogorov–Smirnov, Lilliefors-corrected K–S, and Shapiro–Wilk as principal options for normality assessment [46, 47].
2. **Define variables and PRD categories.** The independent variable was the compression level, expressed as the PRD between original and reconstructed signals. The dependent variables were the band-specific power-of-the-errors values $\mathcal {E}_{i,X}$ derived from the PSD. Comparisons used non-overlapping PRD categories (for example, PRD *≤ 15* vs. *> 15* for EEG; PRD *≤ 1* vs. *> 1* for EMG).
3. **Primary hypothesis test.** Because the comparisons involved independent groups (not paired or repeated measures), we used the Wilcoxon rank-sum (Mann–Whitney U) test to compare $\mathcal {E}_{i,X}$ between PRD categories within each group [48]. Tests were two-sided and interpreted as comparisons of distributions, that is a shift in central tendency, under the assumption of independent observations. In addition, we quantified the magnitude of between-category differences by computing the effect size.
4. **Multiple-comparison correction.** To address testing across the five dependent variables (spectral bands) within each group, we applied a Bonferroni correction to control the family-wise error rate at *α = 0.05* (corrected threshold $\alpha _{\text {corr}} = 0.05/5 = 0.01$) [49].
5. **Software implementation.** Normality was assessed in MATLAB (Statistics and Machine Learning Toolbox; lillietest). Nonparametric comparisons were run in Python (pandas; SciPy ranksums, two-sided), with Bonferroni adjustment applied within each group across the five bands ($\alpha _{\text {corr}} = 0.05/5 = 0.01$) [50].

Numerical values of *Z*, Bonferroni-adjusted *p*-values, and effect sizes *r* for each band and group are reported in the Supplementary Information.

These procedures assume independent observations. Because some recordings contributed multiple segments, we note this as a limitation and used nonparametric methods to reduce sensitivity to distributional assumptions. Although some segments may have originated from the same parent recording, the segments included in the analysis were chosen to cover a broad range of codec-generated distortion levels across diverse signal examples. The resulting comparisons should therefore be interpreted as exploratory between-group analyses rather than as full recording-level population inference. A mixed-effects approach would be better suited to future studies in which each segment’s source recording is tracked explicitly throughout the analysis process.

Consistent with recommended practice, the Lilliefors-corrected Kolmogorov–Smirnov test was used because a plain one-sample K–S test assumes a fully specified reference distribution; when the mean and variance are estimated from the sample, an adjusted null distribution is required [49, 51].

We recognize that the data could also be analyzed with a multifactorial model including a group-by-PRD interaction. For this exploratory study, we did not model such interactions and instead tested whether the distribution (central tendency) of the power-of-the-errors $\mathcal {E}_{i,X}$ differs between PRD categories within each group [49].

## Experimental Results

Through visual inspection, our study suggests that PRD values above approximately 15% for EEG and above approximately 1% for EMG were associated with greater perceived distortion in the evaluated datasets. These observations should be interpreted within the exploratory scope of the study and not as definitive or universally applicable clinical thresholds.

**Fig. 1:**
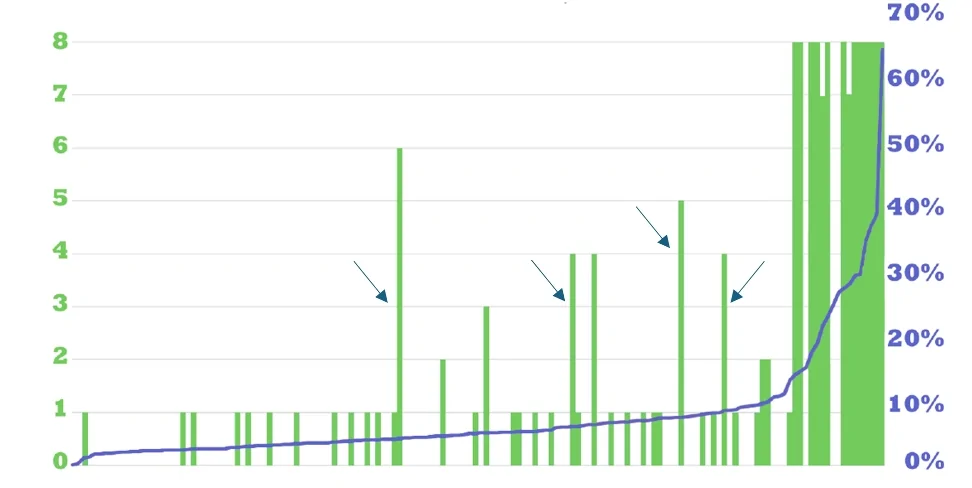
EEGnet output results: the scores for each EEG segment (illustrated by green bars) and their corresponding PRD values (shown by the blue line) (Adapted from Filippo Battaglia, *Neurophysiology Signal Codecs for the DICOM® Standard: Preliminary Results*. Presented at the 2024 IEEE International Symposium on Medical Measurements and Applications (MeMeA); published by IEEE. © Copyright 2025 IEEE - All rights reserved, including rights for text and data mining and training of artificial intelligence and similar technologies. Reprinted with permission from IEEE Proceedings)

Figure 1 presents a detailed summary of the EEGnet expert opinion results for the 150 single-channel MUSC EEG segments, with scores ranging from 0 to 8, represented by green vertical bars. Higher scores indicate a greater number of experts detecting significant compression artifacts. Specifically, a score of 8 signifies that all experts observed substantial artifacts, while a score of 0 means no experts noted significant artifacts. The segments are organized by their PRD values for clarity as depicted by the blue curve. Through subjective visual inspection, our study suggests that a PRD threshold of up to 15% may be acceptable for EEG data.

Several EEG datasets exhibited relatively low PRD values but were nevertheless rated by some experts as showing clinically significant distortion. These cases are indicated by arrows in Fig. 1.

**Fig. 2:**
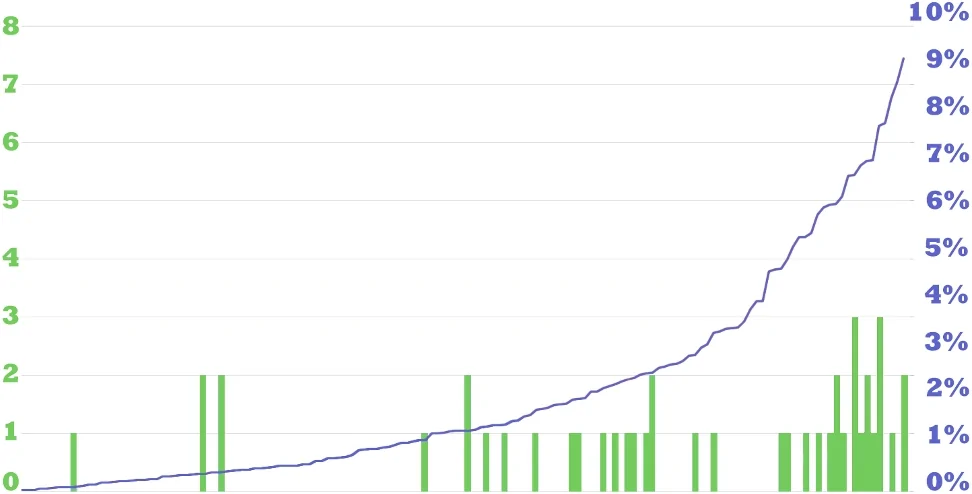
EEGnet output results: the scores for each EMG segment (illustrated by green bars) and their corresponding PRD values (shown by the blue line)

Similarly, Fig. 2 illustrates the analysis of the 145 Mendeley surface EMG segments, following the same structure as Fig. 1. The scores, also ranging from 0 to 8, are shown as green vertical bars, reflecting the number of experts who reported significant artifacts. This result suggests that PRD values around 1% may already affect perceived surface EMG quality in the evaluated dataset; however, because none of the tested PRD levels produced majority expert agreement of clinically significant distortion, this observation should be considered tentative and inconclusive.

**Fig. 3:**
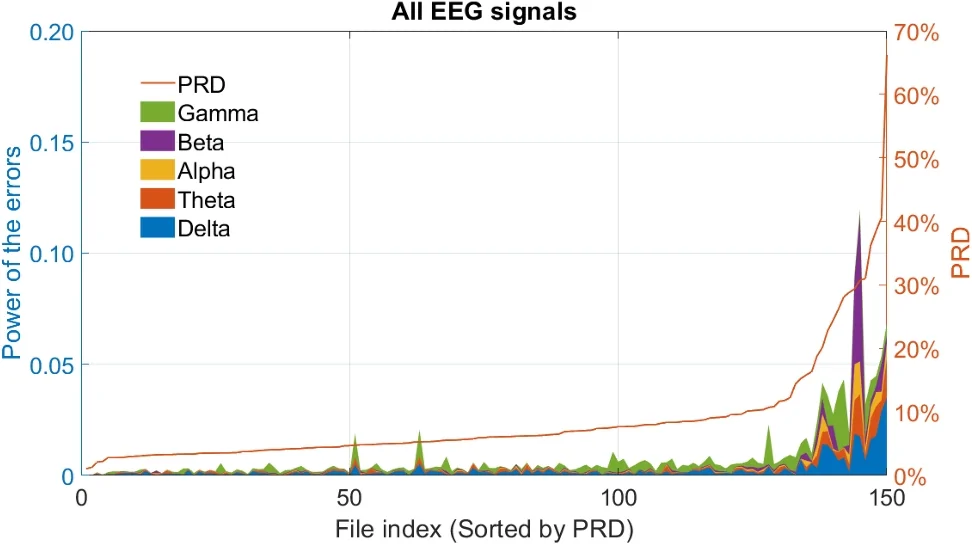
Power of the errors ($\mathcal {E}_{i,X}$) related to all EEG signals

**Fig. 4:**
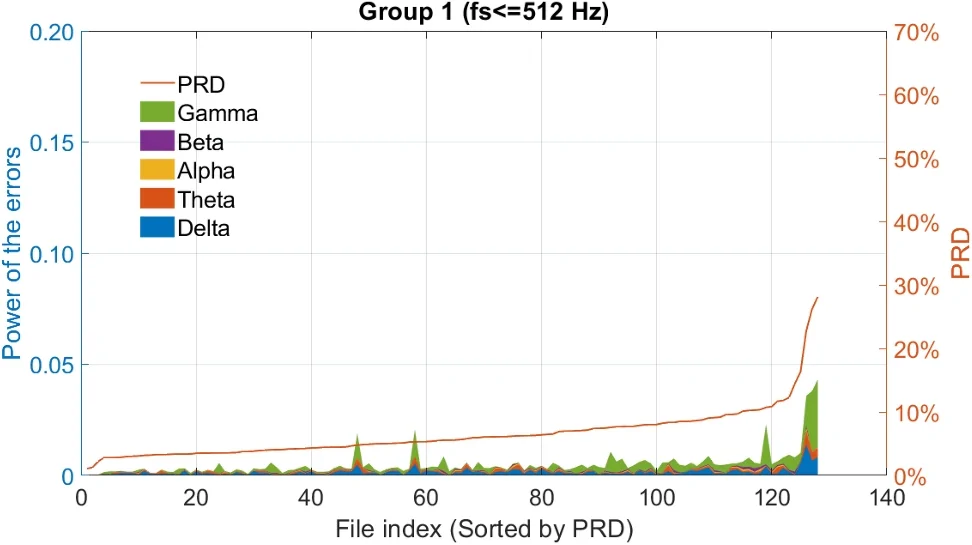
Power of the errors ($\mathcal {E}_{i,X}$) related to EEG signals in Group 1

**Fig. 5:**
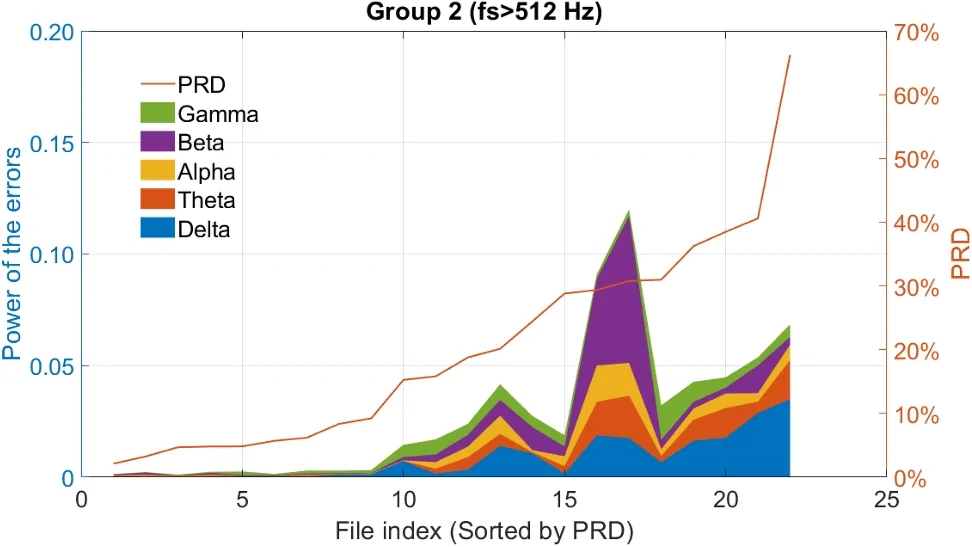
Power of the errors ($\mathcal {E}_{i,X}$) related to EEG signals in Group 2

**Fig. 6:**
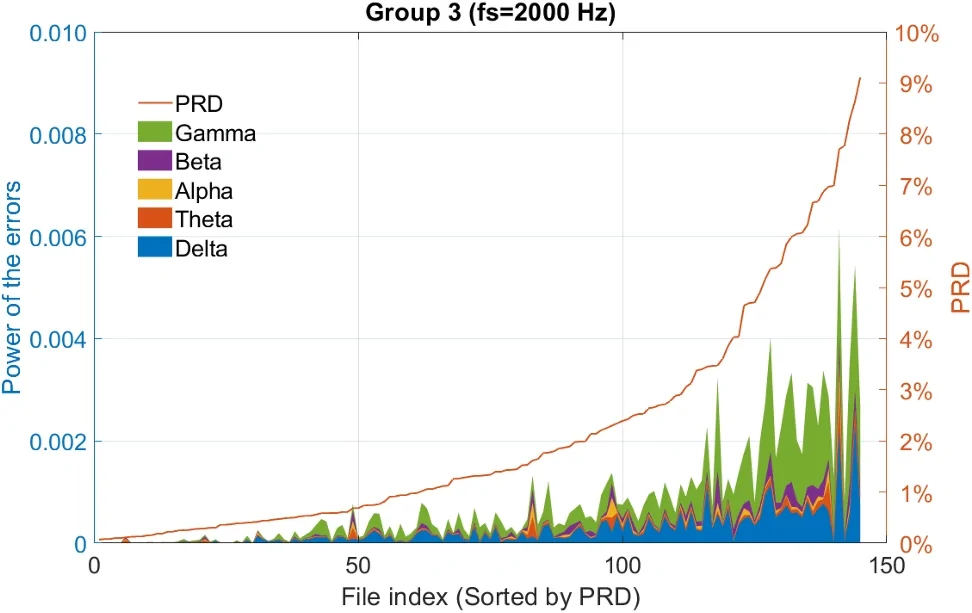
Power of the errors ($\mathcal {E}_{i,X}$) related to EMG signals in Group 3

Figure 3 plots the power of the errors $\mathcal {E}_{i,X}$ related to the EEG signals as well as the corresponding PRD values.

The most significant values in $\mathcal {E}_{i,X}$ that were measured in the spectral bands apparently came from the iEEG signals in Group 2 (see Fig. 5) and not from the scalp EEG signals in Group 1 (see Fig. 4).

The EEG signals in Group 1 experienced the highest power of the errors $\mathcal {E}_{i,X}$ mainly in gamma, beta and theta bands (listed in decreasing order), whereas the EEG signals in Group 2 experienced the highest power of the errors $\mathcal {E}_{i,X}$ in gamma, beta and alpha bands. Figure 6 plots the power of the errors $\mathcal {E}_{i,X}$ in the five spectral bands related to EMG signals in Group 3, as well as the corresponding PRD values. Differences were mainly observed in gamma, beta and alpha bands, for distortion levels above and below the PRD cutoff value of 1%.

The visual patterns in Figs. 3, 4, 5 and 6 are consistent with the results of our formal statistical analysis. Nonparametric comparisons between low- and high-PRD categories (Wilcoxon rank-sum with Bonferroni correction) showed only small-to-moderate effects in a subset of bands for scalp EEG (primarily delta and gamma), more widespread and large effects across all bands in intracranial EEG, and large effects across all bands in EMG. Detailed normality tests, Wilcoxon statistics, Bonferroni-adjusted *p*-values, and effect size estimates (*r*) are reported in the Supplementary Information.

**Fig. 7:**
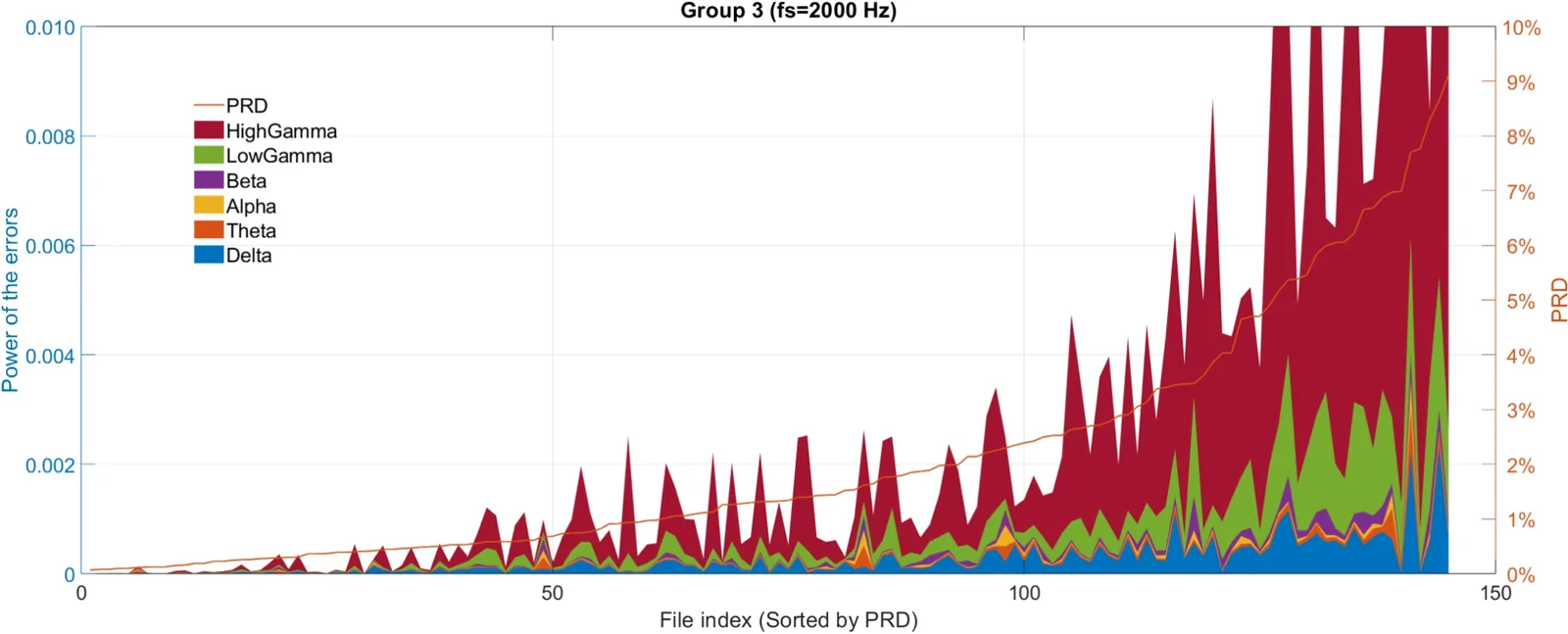
Extended EMG analysis showing the power of the errors $\mathcal {E}_{i,X}$ across six spectral bands, including the additional high-gamma range (100–400 Hz)

To better capture the broader spectral content of EMG signals, we performed an extended analysis by subdividing the gamma range into low-gamma (30–100 Hz) and high-gamma (100–400 Hz) components. This additional evaluation aimed to determine whether higher-frequency muscle activity exhibited disproportionate sensitivity to compression. The results, shown in Fig. 7, confirm that the general distribution of error power remains consistent across PRD levels, while the high-gamma range displays slightly elevated distortion at higher compression ratios.

In this study, formal statistical inference was pre-specified only for the canonical five frequency bands (delta, theta, alpha, beta, and gamma). All normality assessments, Wilcoxon rank-sum tests, Bonferroni corrections, and effect size estimates reported for $\mathcal {E}_{i,X}$ were therefore performed on these canonical bands. The extended low-gamma (30–100 Hz) and high-gamma (100–400 Hz) EMG analysis in Fig. 7 was treated as exploratory and descriptive, and no additional hypothesis tests were specified for these sub-bands.

## Conclusions

### Impact on Signals

The results from EEGNet analysis suggest that EEG signals remain acceptable for most experts with a PRD up to 15%. However, the findings also indicate some limited evidence for decreased quality in surface EMG signals when the PRD exceeds 1%. These modality-specific PRD cutoffs should not be taken to mean that AAC is inherently more faithful for EMG than for EEG. Instead, they likely reflect that experts notice and weigh codec-related changes differently across modalities. For surface EMG, the $\approx 1\%$ PRD value is based on limited evidence and did not produce majority agreement across distortion levels, so we view it as a tentative, hypothesis-generating observation that should be examined further with EMG-relevant endpoints. PSD analysis revealed significant changes in the gamma and beta bands for both EEG and EMG signals. The primary takeaway is that maintaining PRD below 15% is crucial for preserving the PSD of key frequency bands in EEG signals, especially in mixed datasets. For surface EMG signals, the sensitivity to PRD is most pronounced in the gamma band, though the impact on other frequency bands may be less severe, with PRD under 1% acting as a threshold for signal integrity.

This study underscores the importance of PRD management to maintain the integrity of neurophysiological signals, balancing compression with the need to preserve essential signal features. These findings provide guidance for developing compression techniques that prioritize data reduction while ensuring reliable and accurate neurophysiological data for analysis.

Some EEG datasets with relatively low PRD values were still noted by certain experts as showing noticeable or substantial distortion. In contrast, other recordings with higher PRD values showed little or no visible alteration. It is probable that the psychoacoustic masking used by AAC removed weak high-frequency details whenever stronger low-frequency activity was present, making the waveform appear smoother or less detailed to some experts even though the PRD value did not change substantially. Differences in how individual scorers notice and interpret small waveform changes could also have contributed to this variation. We acknowledge that our current approach to collecting expert opinion on waveform distortion from codecs can be improved. In future work, we plan to define “clinically significant distortion” more precisely, use a 5-point Likert scale instead of a binary decision to capture more nuanced ratings, and include tests of experts’ ability to detect clinically relevant abnormalities in addition to providing subjective distortion scores.

### MPEG-4 AAC Compression Algorithm and Its Limitations

This study provides further evidence that MPEG-4 AAC does not adequately support the compression of neurophysiological waveforms. MPEG-4 AAC was chosen for this evaluation due to its reputation for high compression ratios and acceptable audio quality [52, 53], making it a logical candidate to be tested for compressing neurophysiological signals. However, the results indicate that MPEG-4 AAC is insufficient for preserving the integrity of biomedical waveform data, particularly EEG signals, where high-frequency components are vital.

To ensure that band-limited analyses did not overlook full-spectrum spectral effects, we included PSD evaluation of full-spectrum power-of-the-errors comparisons using the same Welch-based method. The full-spectrum results (see Supplementary Information) mirrored the canonical-band findings: minimal error power at low PRD, gradual increases with moderate distortion, and a sharp rise only at higher PRD levels. No additional full-spectrum degradation was evident, confirming that the five-band analysis sufficiently captured the principal spectral behavior of the signals.

MPEG-4 AAC compression uses perceptual coding to target frequencies less perceptible to human hearing, often reducing or eliminating high-frequency data. In neurophysiological signals like EEG, where all frequency components are crucial, this approach compromises the integrity of important diagnostic features. The reduction in high-frequency content, particularly in the alpha, beta, and gamma ranges, may hinder the detection of key neurophysiological events such as epileptiform spikes and seizure onsets, potentially leading to misinterpretation or missed diagnoses [54, 55].

We hypothesize that the distortions observed in EEG and EMG after MPEG-4 AAC compression arise directly from the psychoacoustic models used in standard audio codecs. These models discard or attenuate low-energy or high-frequency components when higher-power low-frequency signals are present and prioritize frequencies within the optimal range for human hearing. As a result, information that is perceptually “irrelevant” for listening to audio signals, but is diagnostically important in biomedical waveforms, can be selectively removed by the codec. Similar effects of audio compression on the loss of biologically relevant features have been reported in other research fields [56].

Regarding codec performance across other EEG paradigms, frequency composition plays a crucial role. In paradigms such as motor imagery, working memory, or cognitive tasks, where the spectral content is typically dominated by sustained oscillatory rhythms and less by abrupt, nonstationary transients, the compression behavior of a psychoacoustic codec may differ somewhat. However, the fundamental limitation remains the same—any EEG pattern that involves a mixture of high-frequency activity in the presence of strong lower-frequency components will be prone to selective attenuation of the high-frequency content due to psychoacoustic masking. The effect would likely lead to the loss of diagnostically or functionally relevant information in clinical EEG, and possibly clinical EMG, recordings.

### Implications for Standardization

The broader motivation for this work stems from international standardization needs rather than algorithmic innovation. The DICOM® standard permits compression only through codecs which are standardized, and therefore maintained by recognized, non-profit international standards organizations such as ITU-T, the International Organization for Standardization (ISO), or the International Electrotechnical Commission (IEC). There is currently no standardized codec for biomedical or general scientific waveform data that can be used as a DICOM® transfer syntax for waveform compression. DICOM® syntaxes are formally registered through the National Electrical Manufacturers Association (NEMA) and must comply with strict interoperability and reliability requirements.

At present, most standardized high-quality audio codecs, including those in the MPEG family such as AAC and High-Efficiency Advanced Audio Coding (HE-AAC), achieve high compression efficiency by exploiting frequency and temporal masking phenomena in human hearing to remove perceptually irrelevant components [16, 57]. While this approach is effective for perceptual audio coding, it inherently discards low-energy or high-frequency components that may be clinically relevant in biomedical recordings. In contrast, several academic approaches to biomedical signal compression, such as wavelet-based, entropy-based, or near-lossless methods, provide good performance but remain unstandardized and non-interoperable within the DICOM® framework.

Within this context, the present study further provides quantitative evidence that an existing, internationally standardized audio codec fails to meet the fidelity requirements for biomedical waveform integrity. The results demonstrate why audio-based compression using psychoacoustic models cannot be adopted for neurophysiological signals and highlights the need for a dedicated, standards-track codec specifically designed for neurophysiology. These considerations have guided the development of ITU-T Recommendation H.BWC, a dedicated codec for biomedical and scientific waveforms that will also probably be standardized as MPEG T.261 (ISO/IEC 23003-8) [58].

### Future Directions

Future research will focus on the analysis of the performance of the upcoming /ISO/IEC T.261 codec. We will also expand the types of clinical neurophysiology signals analyzed to include needle EMG, microelectrode EEG, and a greater amount of iEEG. Needle EMG recordings have higher sampling frequencies and will likely exhibit different morphology and power spectral signatures compared to surface EMG, possibly resulting in a different level of distortion when subjected to lossy compression. Additionally, because iEEG signals demonstrated more distortion than surface EEG signals, future studies will incorporate more iEEG data for a comprehensive assessment of compression methods.

We are currently planning a study to evaluate the T.261 codec, which is designed for biomedical and general scientific waveform encoding. This study will include a statistical plan with power analysis and use a blocked two-way factorial or three-way factorial design, incorporating expert opinion (gathered via EEGnet), PSD features, and other signal analysis techniques. Future work will consider blinding the signal order to further eliminate any potential bias.

The primary objective of this study was to assess whether MPEG-4 AAC compression introduces clinically significant differences in EEG and EMG data that could impact patient care. While not all types of artifacts or distortions were fully characterized, the observed loss of high-frequency content in EEG signals due to compression is a significant finding. This suggests that current audio codec compression techniques which utilize psychoacoustic models do not fully preserve the integrity of neurophysiological signals, emphasizing the need for the upcoming T.261 codec. These findings should be interpreted within the exploratory scope of the present study. Although these findings are dataset-specific and exploratory, they remain relevant to ongoing DICOM® and related standardization efforts because they provide clinically grounded evidence of the limitations of applying a standardized psychoacoustic audio codec to neurophysiological waveforms. The expert-opinion framework used here was intentionally simple and may not capture the full range of perceptual variation across reviewers. For that reason, the reported PRD values are best understood as observations from the present dataset and evaluation setting rather than as definitive or universally applicable clinical thresholds. Future work may benefit from more granular rating schemes and broader validation datasets.

## Supplementary Information

Below is the link to the electronic supplementary material.

Supplementary file 1 (pdf 658 KB)

## Data Availability

De-identified data supporting the findings of this study are available upon reasonable request via email.
