# Supplementary material for: Evaluation of MPEG-4 AAC Audio Codec Using EEG and EMG Signals for Use with DICOM® Neurophysiology
Source: J Med Syst. 2026 Aug 26;50(1):126. doi: 10.1007/s10916-026-02451-9 (PMC13518441; doi:10.1007/s10916-026-02451-9)
Supplement: Supplementary file 1 [file 10916_2026_2451_MOESM1_ESM.pdf]

Supplementary Information

Journal of Medical Systems

Evaluation of MPEG-4 AAC Audio Codec Using EEG and EMG Signals for Use with DICOM® Neurophysiology

Mattia Galanti[1,8*], Filippo Battaglia[2], Giovanni Gugliandolo[2], Petter M. Omland[3], Kristian B. Nilsen[4], Sarah Breevoort[6], M. Noreen Herring[5], Jerome Kurent[6,5], I-Hweii A. Chen[7], Tuan Vu[7], Kyle Wuthrich[7], Nicola Donato[2], Mulugeta Gebregziabher[8,5], Giuseppe Campobello[2], Brian C. Dean[1], Jonathan J. Halford[5]

[1*]School of Computing, Clemson University, Clemson, 29631, SC, USA.
[2]Department of Engineering, University of Messina, Messina, 98166, Italy.
[3]Department of Neuromedicine and Movement Science, Faculty of Medicine and Health Sciences, Norwegian University of Science and Technology, Trondheim, N-7491, Norway.
[4]Department of Neurology, Section of Clinical Neurophysiology, Oslo University Hospital, Oslo, Norway.
[5]Ralph H. Johnson Veterans Affairs Medical Center, Charleston, 29401, SC, USA.
[6]Department of Neurology, Medical University of South Carolina, Charleston, 29425, SC, USA.
[7]Morsani College of Medicine, University of South Florida, Tampa, 33602, FL, USA.
[8]Department of Public Health Sciences, Medical University of South Carolina, Charleston, 29425, SC, USA.

*Corresponding author(s). E-mail(s): mgalant@clemson.edu;
Contributing authors: fibattaglia@unime.it; giogugliandolo@unime.it; petter.m.omland@ntnu.no; k.b.nilsen@medisin.uio.no; breevoor@musc.edu; mary.herring@va.gov; kurentje@musc.edu; cheni20@usf.edu; tvu6@usf.edu; kylewuthrich@usf.edu; ndonato@unime.it; gebregz@musc.edu; giuseppe.campobello@unime.it; bcdean@clemson.edu; Jonathan.Halford@va.gov;

Datasets and Codec Application

The electroencephalography (EEG) dataset consists of de-identified, approximately 10-minute excerpts from 41 recordings obtained from the Medical University of South Carolina (MUSC) using Natus equipment. This collection of EEGs comprises excerpts from 31 scalp EEGs recorded with the 10-20 electrode system [1] and Ag/AgCl surface electrodes and 10 excerpts from intracranial electroencephalograms (iEEGs) from depth electrodes, each featuring around 100 channels. All recordings contain interictal epileptiform discharges (IEDs) or electrographic seizures, with sampling rates ranging between 256 Hz and 2.048 kHz. For this study, 150 one-minute segments were selected and extracted from these MUSC recordings by one of the co-authors. The signals were chosen to contain either a seizure or an IED in a salient EEG channel in a broad range of codec-generated distortion levels. Although the dataset consists of 10-minute excerpts from 41 recordings—amounting to over 41,000 one-minute segments—it was not feasible to ask the experts to review all of them, as this would be too time-consuming. Instead, we limited the number of segments to a reasonable amount, observing that in similar studies on electrocardiography (ECG) signals, the number of segments investigated is in the order of 100 [2–4].

The electromyography (EMG) dataset consists of multichannel surface electromyography (sEMG) recordings from four forearm muscles of 40 subjects, originating from a human-computer interaction study found in the Mendeley database and freely available on the web [5]. These recordings were made using a BIOPAC MP36 device with Ag/AgCl surface bipolar electrodes [6]. One of the co-authors chose 145 single-channel signals from these recordings so as to cover a broad range of codec-generalized distortion levels.

The biometric data were processed using the implementation of the Advanced Audio Coding (AAC) encoder released by the Fraunhofer Institute for Integrated Circuits (Fraunhofer IIS) [7], hereinafter named FDK AAC. FDK AAC compression performance largely depends on the parameter named "–bitrate-mode" (hereinafter shortened as b) that is an integer value in the range [1..5]. Usually, lower values of b determine higher compression ratios at the cost of higher distortion values.

Another parameter that affects compression results and was investigated in our experiments is the sampling rate. EEG and EMG signals are typically sampled at lower frequencies, ranging from 256 Hz to 2 kHz. However, audio codecs like the Moving Picture Experts Group version 4 Advanced Audio Coding (MPEG-4 AAC) require a minimum sampling rate of 8 kHz for proper functionality. To address this issue, we specified fake compression frequencies—8 kHz, 16 kHz, and 32 kHz—within the headers of Waveform Audio File Format (WAV) files before compression. This workaround allowed the codec, optimized for higher-frequency audio signals, to process the neurophysiological data despite the mismatch in sampling rates. While this solution enabled meaningful analysis of the codec's performance, it introduced potential limitations, as the codec's algorithms, tuned for audio, may not have performed optimally with neurophysiological signals. This discrepancy could affect compression efficiency and signal quality, potentially introducing distortions or artifacts in the compressed signals. Exploring resampling or alternative codecs could address these limitations, but both would expand the study's scope. Therefore, using fake frequencies was a necessary and pragmatic solution to investigate the MPEG-4 AAC codec's applicability to neurophysiological signals, though it underscores the need for codecs designed specifically for biomedical data.

EEGnet Scoring

Experts were given control of high-pass, low-pass, and notch filtering. By time-synchronizing the display of the signal pairs, experts could scroll through and compare waveform morphology between original and compressed/decompressed waveforms carefully. Building on these user-centric features, the latest version of EEGnet—EEGnet 3.0—was engineered as a dependable platform for real-time assessment of EEG, EMG, and other biomedical signals. EEGnet 3.0 runs under Ubuntu Linux and is hosted on Google Cloud Platform (GCP). The application includes a WebSocket coded in C++ to facilitate rapid real-time data transmission. The frontend is written in Javascript and the backend is powered by a MySQL database [8, 9].

The decision to display the original signal on top and the compressed/decompressed signal below was made for two primary reasons. First, the study aimed to assess whether the signals were practically identical or exhibited clinically significant degradation, and it was assumed that knowledge of signal order would not influence expert assessments. Second, maintaining a consistent top-bottom display order minimized the software development effort required for the EEGnet web-based review

system by avoiding the need for randomized signal placement. While this approach introduced the potential for bias, it was deemed sufficient for the purposes of this study.

Experts cast their Yes/No votes via the EEGnet interface (as seen in Fig. 1 for EEG scoring and Fig. 2 for EMG scoring). All EEG signals were evaluated by all eight of the EEG experts, who were all neurologists with fellowship training in EEG interpretation. All EMG signals were evaluated by all eight EMG experts, who were all neurologists with fellowship training in the neuromuscular subspecialty.

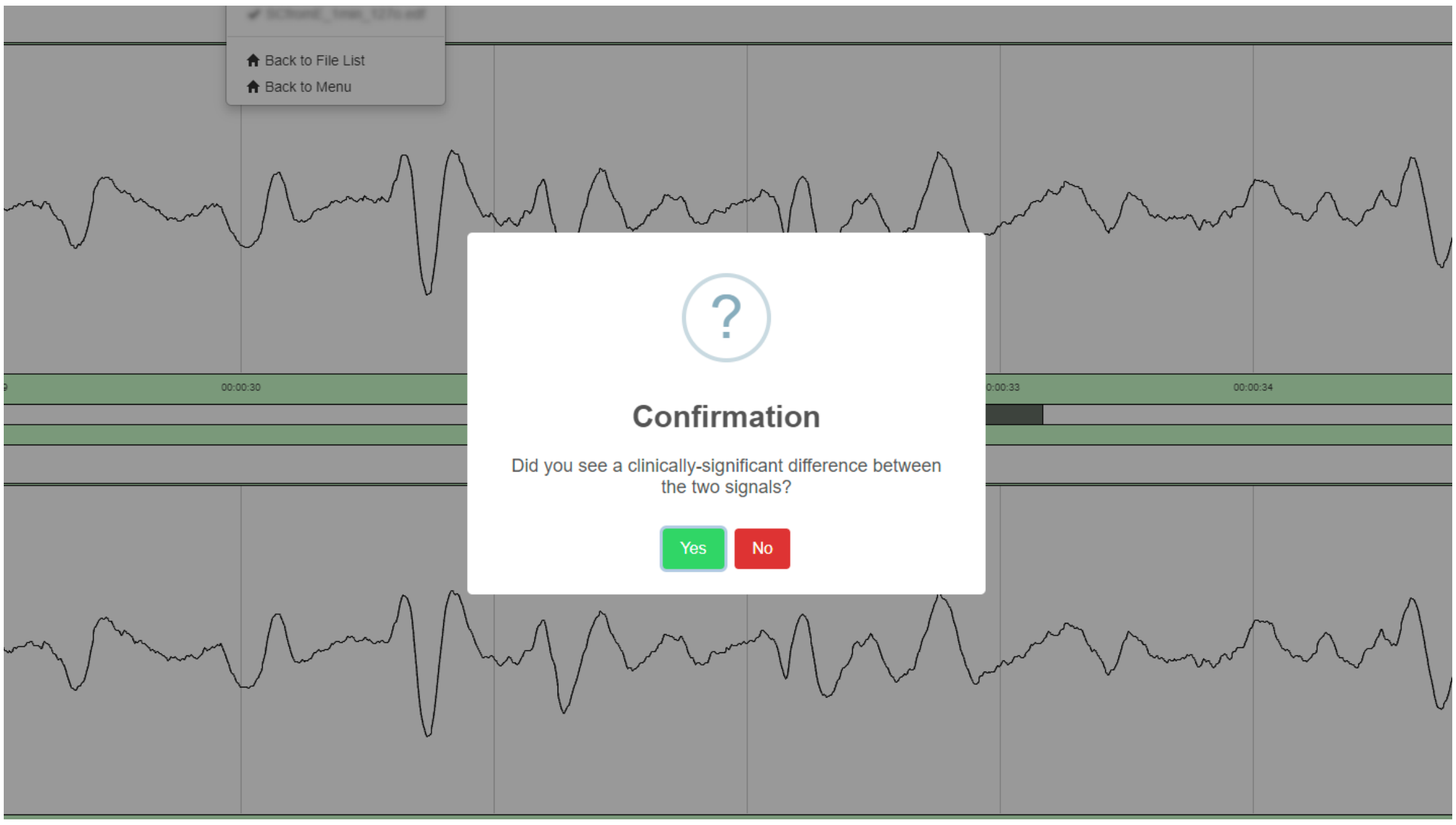

Fig. 1 EEGnet interface: scoring of EEG signals

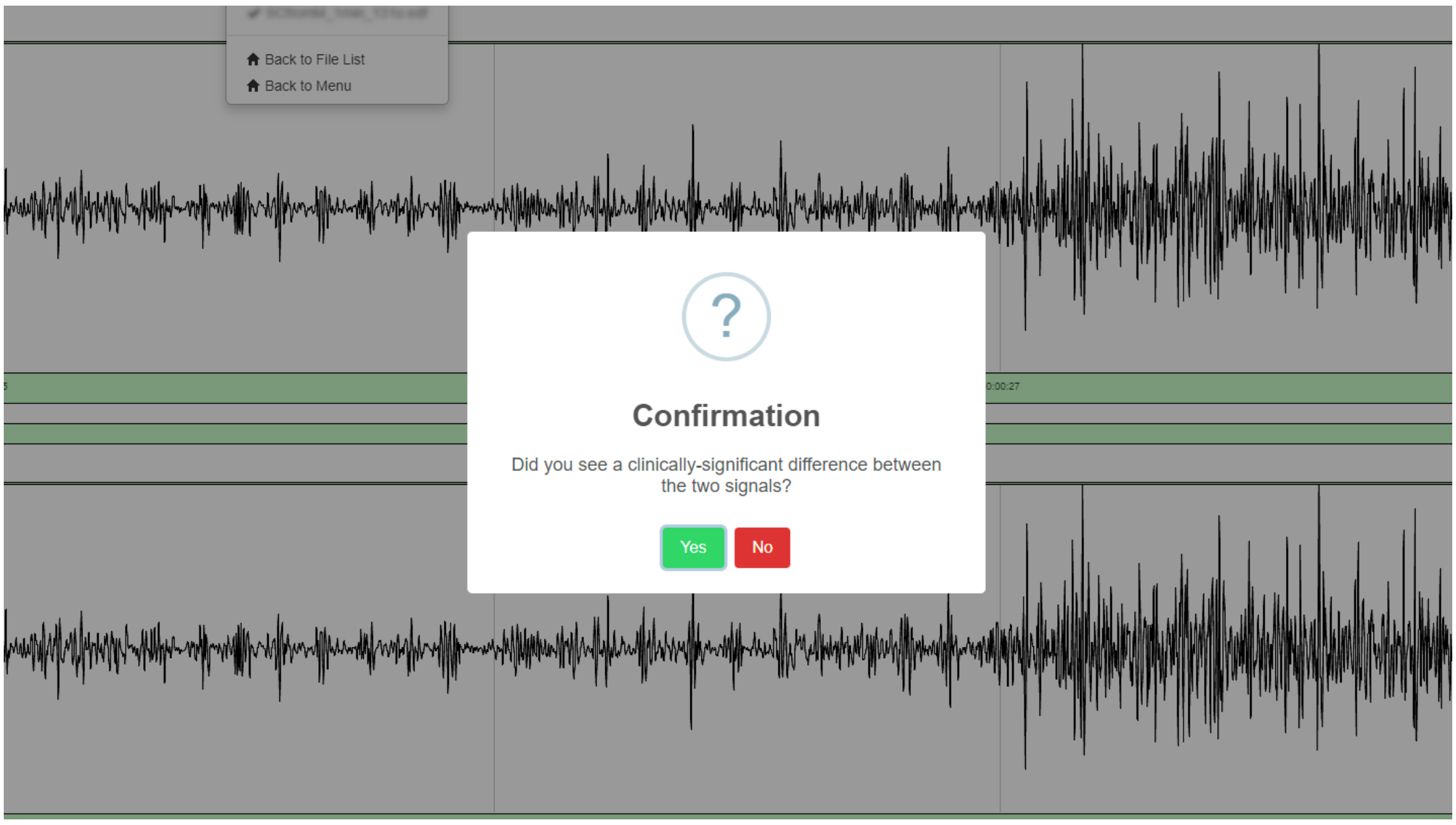

Fig. 2 EEGnet interface: scoring of EMG signals

Distortion Metrics

The power spectrum was estimated using the *pwelch()* MATLAB function [10, 11] with a Hanning window and 50% overlap as follows:

$$PSD_i = pwelch\{s_i, hann(w), w/2, n_{FFT}, f_s\} \tag{1}$$

where:

- s_i is the processed signal;
- n_{FFT} is the number of FFT points used to evaluate the spectral density;
- f_s is the sampling frequency;
- w is the length of the Hanning window.

In our experiments we fixed both the length of the Hanning window and the number of FFT points equal to $2f_s$, i.e. $n_{FFT} = w = 2f_s$. As a consequence, considering that f_s is the number of samples per second of a signal, samples are processed with a time window of 2 seconds and the expected frequency resolution is equal to 0.5 Hz.

The power spectral density (PSD) values have been normalized with respect to the overall power of the signal, thus obtaining the normalized PSD, $NPSD_i = \frac{PSD_i}{P_i}$, where $P_i = \sum_k PSD_i(k)$.

In the same manner we evaluated the normalized power spectra of the reconstructed signal, $NPSD_i^*$.

Finally, we calculated the power of the errors for each of the five spectral bands as follows:

$$\mathcal{E}_{i,X} = \sum_{k \in \mathcal{R}_X} |NPSD_i(k) - NPSD_i^*(k)| \tag{2}$$

where $X \in \{alpha, beta, gamma, delta, theta\}$ and $\mathcal{R}_X$ is the subset of FFT bins corresponding to the X bandwidth.

Full-Spectrum Analysis

In addition to the five canonical frequency bands, a complementary full-spectrum analysis was conducted to determine whether compression artifacts extended beyond conventional neurophysiological ranges. This assessment employed the same Welch-based PSD method and power-of-the-errors formulation described above but was applied across the entire frequency spectrum rather than within predefined sub-bands.

The resulting full-spectrum power-of-the-errors curves for EEG (Fig. 3 – 5) and EMG (Fig. 6) confirmed that distortion behavior across the full spectrum closely paralleled the canonical-band findings. Error power remained minimal at low Percentage Root Mean Square Difference (PRD) values, increased gradually with moderate distortion, and rose sharply only at higher compression levels. This pattern was consistent across pooled EEG data as well as scalp-dominant, intracranial-dominant, and surface EMG subsets. No additional full-spectrum degradation was observed at low PRD, supporting that the five-band analysis reported in the main manuscript adequately represented the principal spectral effects of compression.

All full-spectrum analyses were performed in MATLAB using the same data segments, FFT configuration, windowing, and averaging parameters described for the canonical-band PSD computation.

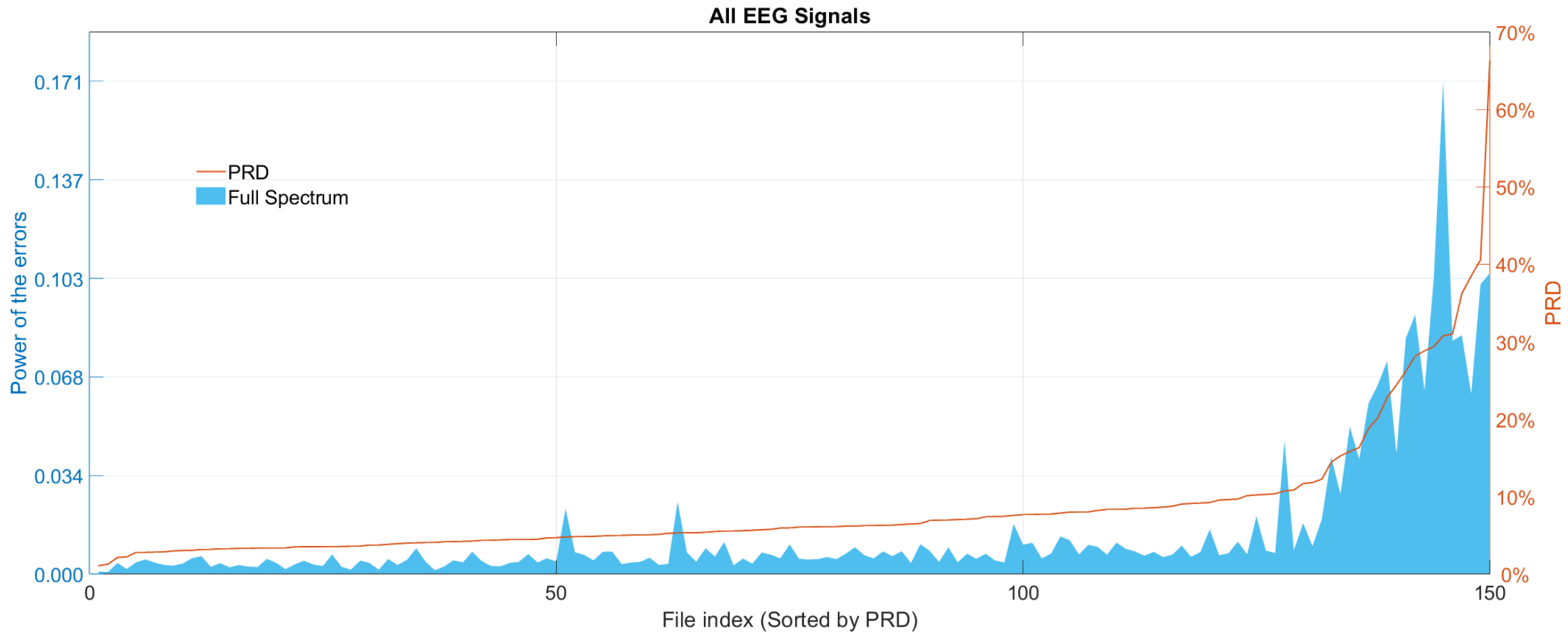

Fig. 3 Full-spectrum power of the errors $(\mathcal{E}_{i,X})$ related to all EEG signals

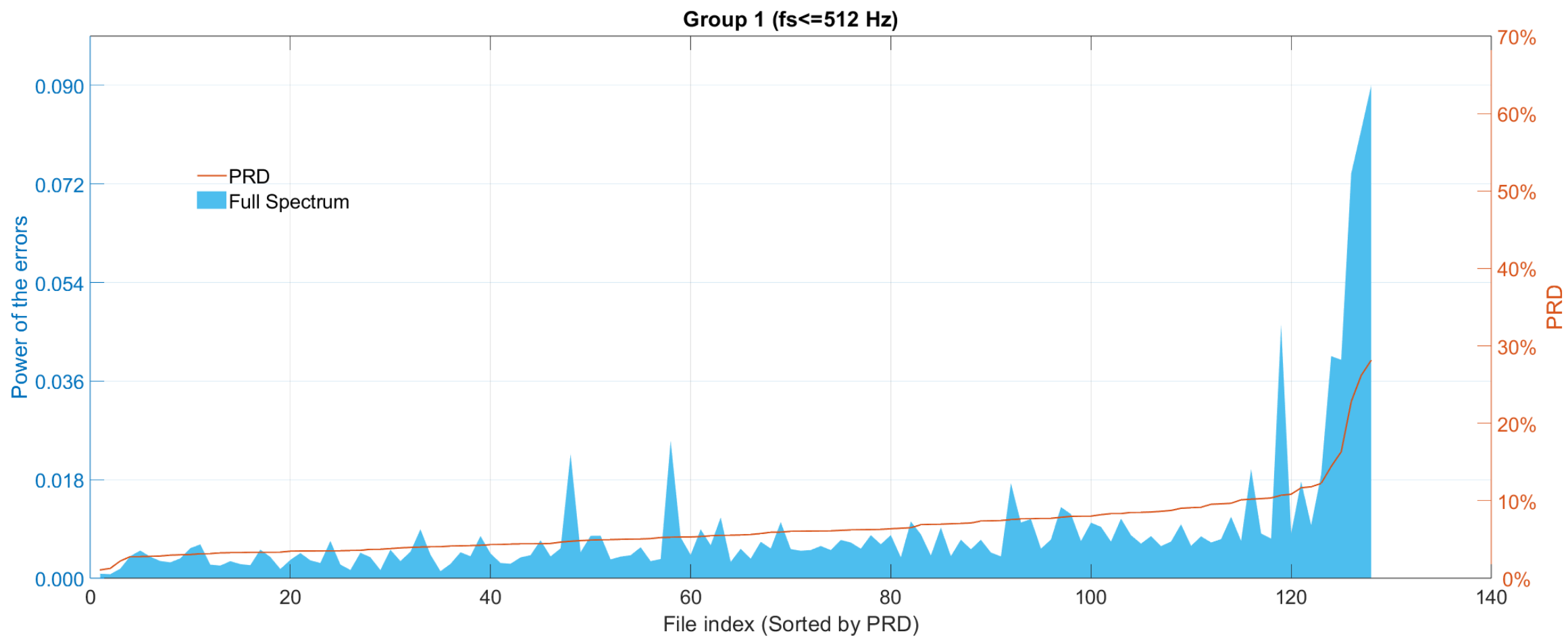

Fig. 4 Full-spectrum power of the errors $(\mathcal{E}_{i,X})$ related to EEG signals in Group 1

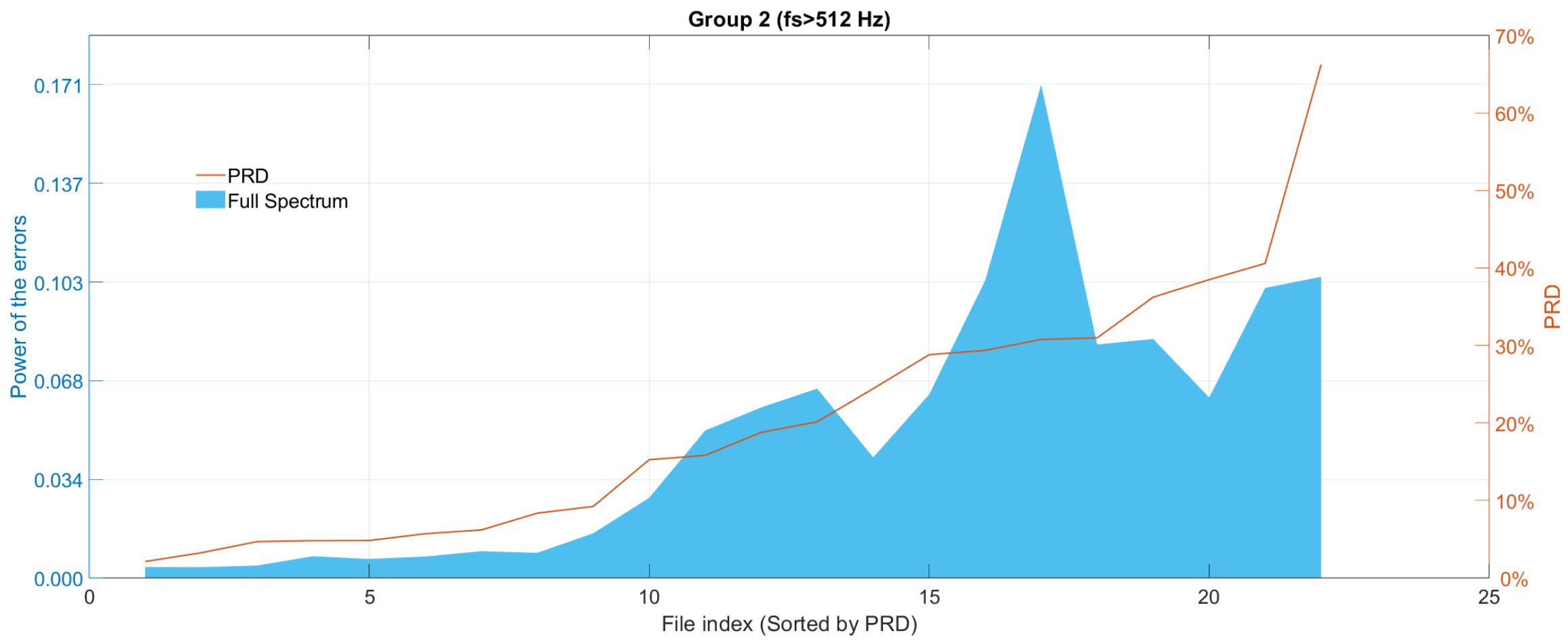

Fig. 5 Full-spectrum power of the errors $(\mathcal{E}_{i,X})$ related to EEG signals in Group 2

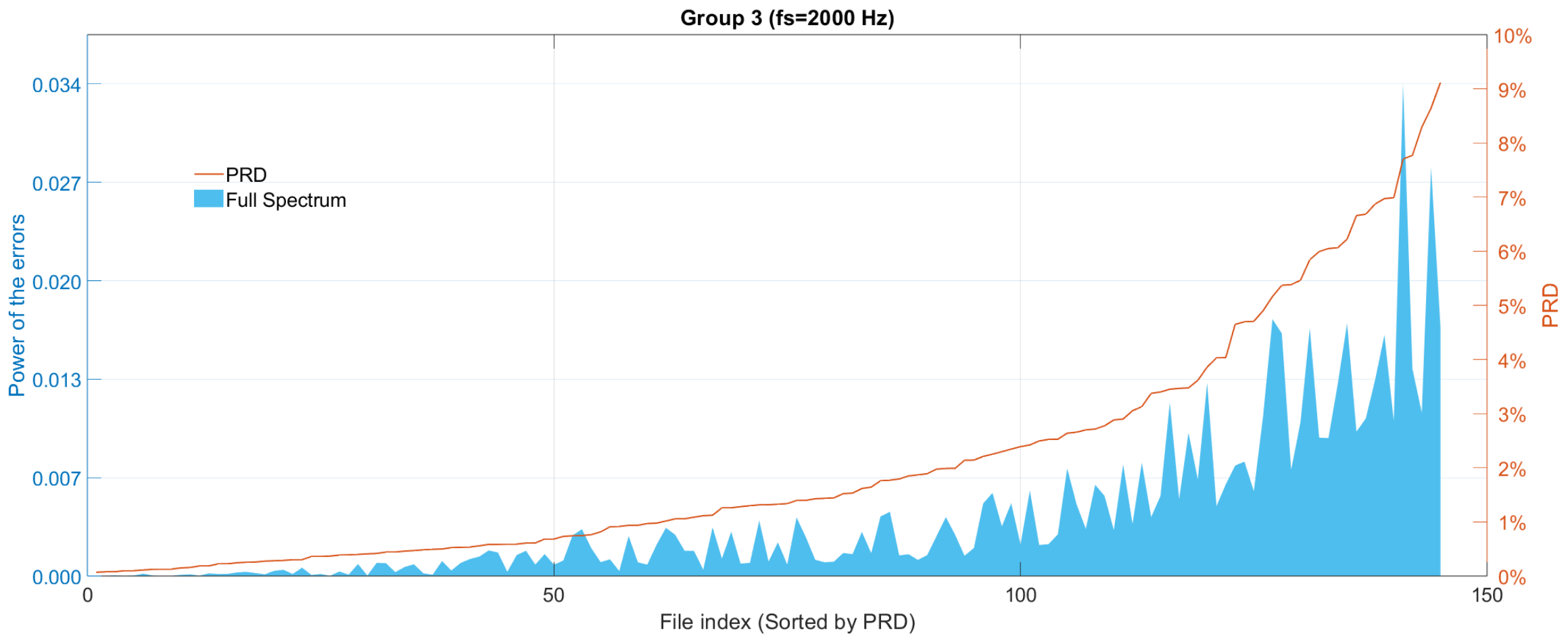

Fig. 6 Full-spectrum power of the errors ($\mathcal{E}_{i,X}$) related to EMG signals in Group 3

Statistical Analysis

Normality Assessment

As described in the Statistical Analysis subsection of the main manuscript, normality of the power-of-the-errors distributions $\mathcal{E}_{i,X}$ was assessed for each group and spectral band using the Lilliefors-corrected Kolmogorov–Smirnov test (MATLAB `lillietest` [12], $\alpha = 0.05$).

Table 1 reports the test decision (h) and p-values for all groups and bands. A value of $h = 1$ indicates rejection of normality at the 5% level, whereas $h = 0$ indicates that normality could not be rejected [12]. For highly non-normal samples, MATLAB `lillietest` returns a lower-bound value of $p = 0.0010$, which should be interpreted as $p < 0.001$.

Table 1 Lilliefors-corrected Kolmogorov–Smirnov test results for each spectral band and group

Group	Spectral band	h (0 = normal, 1 = non-normal)	p-value
Group 1	Delta	1	0.0010
Group 1	Theta	1	0.0010
Group 1	Alpha	1	0.0010
Group 1	Beta	1	0.0010
Group 1	Gamma	1	0.0010
Group 2	Delta	1	0.0012
Group 2	Theta	1	0.0015
Group 2	Alpha	1	0.0056
Group 2	Beta	1	0.0010
Group 2	Gamma	0	0.1640
Group 3	Delta	1	0.0010
Group 3	Theta	1	0.0010
Group 3	Alpha	1	0.0010
Group 3	Beta	1	0.0010
Group 3	Gamma	1	0.0010

Across groups, 14 out of 15 band–group combinations showed significant deviations from normality. All five canonical bands in Groups 1 and 3 were non-normal ($h = 1$, $p < 0.001$), and in Group 2, four bands (delta, theta, alpha, and beta) were non-normal while the gamma band did not significantly differ from a normal distribution ($h = 0$, $p = 0.1640$). These findings support the use of nonparametric methods for subsequent comparisons of $\mathcal{E}_{i,X}$ between PRD categories.

Wilcoxon Rank-Sum Comparisons for Canonical Bands

Following the analysis plan in the Statistical Analysis subsection of the main manuscript, we compared the distributions of $\mathcal{E}_{i,X}$ between predefined PRD categories within each group using the Wilcoxon rank-sum (Mann–Whitney U) test, with Bonferroni correction across the five canonical bands (corrected threshold $\alpha_{\text{corr}} = 0.01$ per group). Effect sizes were computed as [13]:

$$r = \frac{|Z|}{\sqrt{N}} \tag{3}$$

where:

- Z is the standardized test statistic from the Wilcoxon rank-sum (Mann–Whitney U) test;
- $N = n_1 + n_2$ is the total number of observations across the two PRD categories;
- r is the effect size, interpreted analogously to a correlation coefficient (small: $|r| \approx 0.1$, medium: $|r| \approx 0.3$, large: $|r| \geq 0.5$).

Table 2 reports the Wilcoxon Z-statistics, original p-values, Bonferroni-corrected p-values, and corresponding effect sizes r.

Table 2 Wilcoxon rank-sum test results for power-of-the-errors $\mathcal{E}_{i,X}$ across PRD categories for each spectral band and group

Group	Spectral band	Z-score	p-value	Corrected p-value	Effect size r
Group 1	Delta	-3.396	0.001	0.003	0.300
Group 1	Theta	-2.465	0.014	0.068	0.218
Group 1	Alpha	-1.739	0.082	0.410	0.154
Group 1	Beta	-0.835	0.404	1.000	0.074
Group 1	Gamma	-3.122	0.002	0.009	0.276
Group 2	Delta	-3.907	0.000	0.000	0.833
Group 2	Theta	-3.306	0.001	0.005	0.705
Group 2	Alpha	-3.706	0.000	0.001	0.790
Group 2	Beta	-3.907	0.000	0.000	0.833
Group 2	Gamma	-3.907	0.000	0.000	0.833
Group 3	Delta	-8.495	0.000	0.000	0.705
Group 3	Theta	-8.367	0.000	0.000	0.695
Group 3	Alpha	-8.226	0.000	0.000	0.683
Group 3	Beta	-8.679	0.000	0.000	0.721
Group 3	Gamma	-8.927	0.000	0.000	0.741

In Group 1 (scalp EEG), only the delta and gamma bands remained statistically significant after Bonferroni correction (corrected $p = 0.003$ and 0.009, respectively), with small-to-moderate effect sizes ($r \approx 0.30$ and 0.28). Theta, alpha, and beta were not significant after correction, and their effect sizes were small ($r < 0.25$), indicating only modest shifts in the distribution of $\mathcal{E}_{i,X}$ across PRD categories in these bands.

In Group 2 (intracranial EEG), all five bands (delta, theta, alpha, beta, and gamma) showed statistically significant differences between low- and high-PRD categories after correction (corrected $p \leq 0.005$), with large effect sizes ($r \approx 0.70$–0.83). This pattern indicates a pervasive and pronounced spectral sensitivity to compression in intracranial recordings.

In Group 3 (EMG), all bands exhibited highly significant differences (corrected $p < 0.001$) with large effect sizes ($r \approx 0.68$–0.74), consistent with strong, broadband sensitivity of surface EMG spectra to increasing PRD.

References for Supplementary Information

[1] Jasper, H.H.: Ten-twenty electrode system of the international federation. Electroencephalogr Clin Neurophysiol **10**, 371–375 (1958)

[2] Abo-Zahhad, M.M., Hussein, A.I., Mohamed, A.M., El-Sawy, A.M.: A DEDICATED DIAGNOSTIC DISTORTION MEASURE FOR THE EVALUATION OF ECG SIGNAL COMPRESSION TECHNIQUES ADOPTED FOR REMOTE DIAGNOSIS OF CARDIOVASCULAR DISEASES. Journal of Engineering Sciences **44**(2), 158–174 (2016) https://doi.org/10.21608/jesaun.2016.117594

[3] Manikandan, M.S., Dandapat, S.: Wavelet energy based diagnostic distortion measure for ECG. Biomedical Signal Processing and Control **2**(2), 80–96 (2007) https://doi.org/10.1016/j.bspc.2007.05.001

[4] Alesanco, A., García, J.: Automatic Real-Time ECG Coding Methodology Guaranteeing Signal Interpretation Quality. IEEE Transactions on Biomedical Engineering **55**(11), 2519–2527 (2008) https://doi.org/10.1109/TBME.2008.2001263

[5] Ozdemir, M.A., Kisa, D.H., Guren, O., Akan, A.: Dataset for multi-channel surface electromyography (sEMG) signals of hand gestures. Mendeley Data, Version 2 (2021). https://doi.org/10.17632/ckwc76xr2z.2

[6] Ozdemir, M.A., Kisa, D.H., Guren, O., Akan, A.: Dataset for multi-channel surface electromyography (sEMG) signals of hand gestures. Data in Brief **41**, 107921 (2022) https://doi.org/10.1016/j.dib.2022.107921

[7] FDK AAC codec. Fraunhofer IIS (2018). https://manpages.ubuntu.com/manpages/focal/man1/fdkaac.1.html

[8] Halford, J.J., Pressly, W.B., Benbadis, S.R., Tatum, W.O.I., Turner, R.P., Arain, A., Pritchard, P.B., Edwards, J.C., Dean, B.C.: Web-Based collection of expert opinion on routine scalp EEG: software development and interrater reliability. Journal of Clinical Neurophysiology **28**(2), 178–184 (2011) https://doi.org/10.1097/WNP.0b013e31821215e3

[9] Halford, J.J., Schalkoff, R.J., Zhou, J., Benbadis, S.R., Tatum, W.O., Turner, R.P., Sinha, S.R., Fountain, N.B., Arain, A., Pritchard, P.B., Kutluay, E., Martz, G., Edwards, J.C., Waters, C., Dean, B.C.: Standardized database development for EEG epileptiform transient detection: EEGnet scoring system and machine learning analysis. Journal of Neuroscience Methods **212**(2), 308–316 (2013) https://doi.org/10.1016/j.jneumeth.2012.11.005

[10] Welch, P.: The Use of fast fourier transform for the estimation of power spectra: a method based on time averaging over short, modified periodograms. IEEE Transactions on Audio and Electroacoustics **15**(2), 70–73 (1967) https://doi.org/10.1109/TAU.1967.1161901

[11] The MathWorks, Inc.: pwelch: Welch Power Spectral Density Estimate. MathWorks Documentation (2021). https://www.mathworks.com/help/signal/ref/pwelch.html

[12] The MathWorks, Inc.: lillietest: Lilliefors Test for Normality. The MathWorks, Inc., Natick, MA, USA (2025). The MathWorks, Inc.. MATLAB function `lillietest` in Statistics and Machine Learning Toolbox. https://www.mathworks.com/help/stats/lillietest.html

[13] Tomczak, M., Tomczak, E.: The need to report effect size estimates revisited. An overview of some recommended measures of effect size. Trends in Sport Sciences **21**(1), 19–25 (2014)
